# Validating Linear Systems Analysis for Laminar fMRI: Temporal Additivity for Stimulus Duration Manipulations

**DOI:** 10.1007/s10548-020-00808-y

**Published:** 2020-11-18

**Authors:** Jelle A. van Dijk, Alessio Fracasso, Natalia Petridou, Serge O. Dumoulin

**Affiliations:** 1 Spinoza Centre for Neuroimaging, Amsterdam, The Netherlands; 2 Experimental Psychology, Utrecht University, Utrecht, The Netherlands; 3 Institute of Neuroscience and Psychology, University of Glasgow, Glasgow, G12 8QB UK; 4 Radiology Department, Imaging Division, Center for Image Sciences, University Medical Center Utrecht, Utrecht, The Netherlands; 5 Experimental and Applied Psychology, VU University, Amsterdam, The Netherlands

**Keywords:** Cortical laminae, fMRI, Visual system, Neuroimaging, Linear systems

## Abstract

Advancements in ultra-high field (7 T and higher) magnetic resonance imaging (MRI) scanners have made it possible to investigate both the structure and function of the human brain at a sub-millimeter scale. As neuronal feedforward and feedback information arrives in different layers, sub-millimeter functional MRI has the potential to uncover information processing between cortical micro-circuits across cortical depth, i.e. laminar fMRI. For nearly all conventional fMRI analyses, the main assumption is that the relationship between local neuronal activity and the blood oxygenation level dependent (BOLD) signal adheres to the principles of linear systems theory. For laminar fMRI, however, directional blood pooling across cortical depth stemming from the anatomy of the cortical vasculature, potentially violates these linear system assumptions, thereby complicating analysis and interpretation. Here we assess whether the temporal additivity requirement of linear systems theory holds for laminar fMRI. We measured responses elicited by viewing stimuli presented for different durations and evaluated how well the responses to shorter durations predicted those elicited by longer durations. We find that BOLD response predictions are consistently good predictors for observed responses, across all cortical depths, and in all measured visual field maps (V1, V2, and V3). Our results suggest that the temporal additivity assumption for linear systems theory holds for laminar fMRI. We thus show that the temporal additivity assumption holds across cortical depth for sub-millimeter gradient-echo BOLD fMRI in early visual cortex.

**Electronic supplementary material:**

The online version of this article (10.1007/s10548-020-00808-y) contains supplementary material, which is available to authorized users.

## Introduction

Magnetic resonance imaging (MRI) is the dominant method to non-invasively study the structure and function of the living human brain. Over the past two decades, this imaging method has seen rapid development, resulting in ever-increasing technical possibilities and applications. With the advent of ultra-high field (7 T and higher) MRI scanners, it has become possible to investigate both structure and function of the human brain at a sub-millimeter resolution (see e.g. De Martino et al. 2018; Dumoulin et al. 2018; Fracasso et al. 2016b, c; Petridou and Siero 2017; Stephan et al. 2019). As the human cortex has a thickness of 1.5 to 4 mm (Fischl and Dale 1999) and is generally comprised of six anatomically (and functionally) distinct laminae or layers (Brodmann 1909, 1903; Gennari 1782; Vogt and Vogt 1919; Vogt 1910), sub-millimeter resolutions enable the investigation of cortical depth-dependent signals reflecting contributions from different cortical layers, also known as laminar MRI. Sub-millimeter functional MRI (fMRI) promises to complement anatomical measurements across cortical layers, adding valuable information about fundamental processing in cortical micro-circuits, and functional properties that reflect feedforward and feedback interactions within the thickness of the cortex (for reviews, see e.g. De Martino et al. 2018; Dumoulin et al. 2018; Lawrence et al. 2017; Petridou and Siero 2017; Self et al. 2019; Self and Roelfsema 2017; Stephan et al. 2019).

**Fig. 1:**
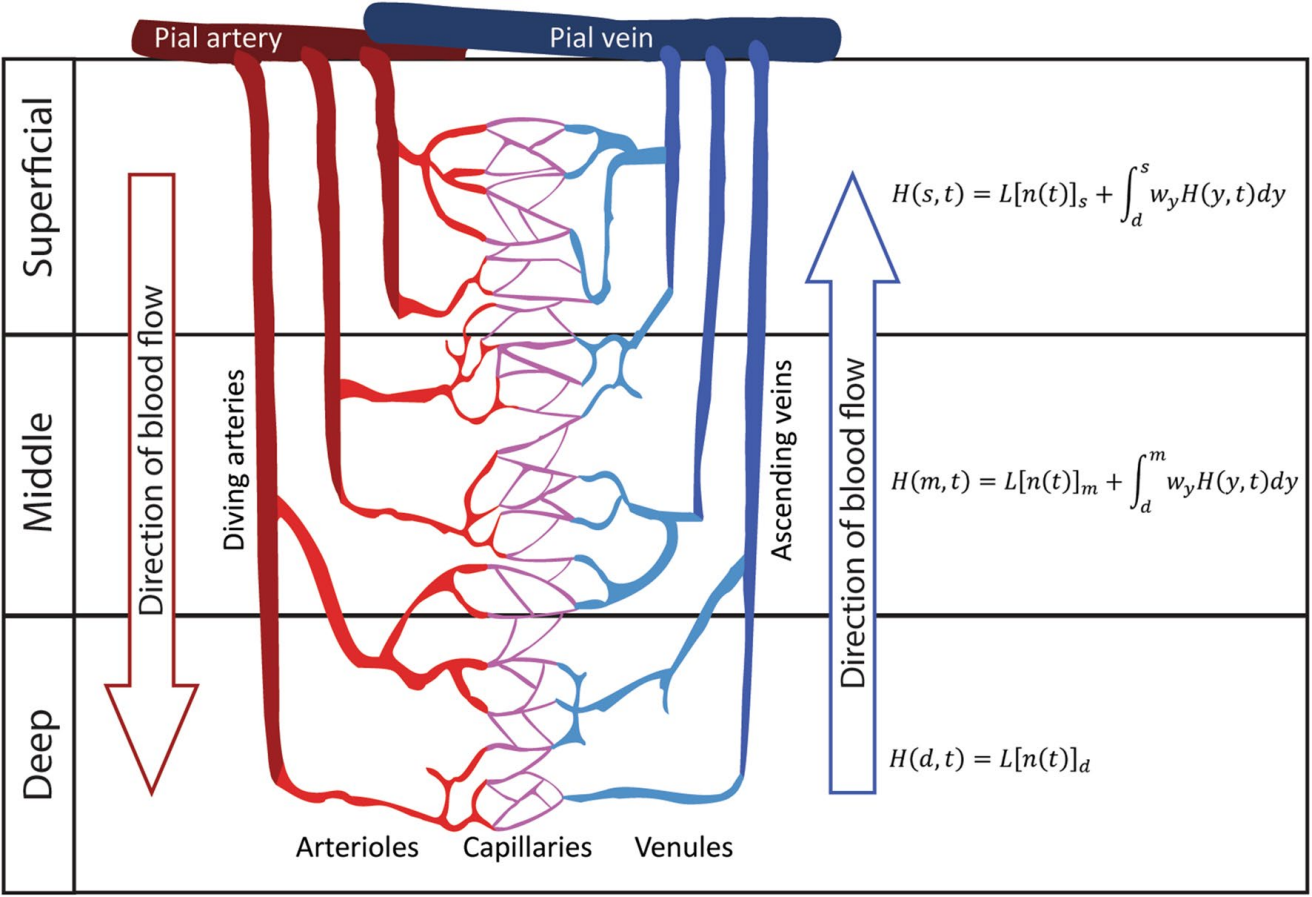
Schematic representation of the cortical vascular organization. Deep (d), Middle (m), and Superficial (s) represent three (arbitrary) cortical depths. Red vasculature represents blood inflow towards the white matter boundary. Blue vasculature represents blood flow towards the cortical surface. Formulae at each depth represent the measured BOLD signal at said depth (see Eq. 2). This signal depends on both the local activity at that depth (${L\left[n\left(t\right)\right]}_{y}$) and vascular consequences of activity at deeper depths, as carried by the directional blood flow (${\int }_{{y}_{0}}^{y}{w}_{y}H\left(y,t\right)dy)$. Figure based on Havlicek and Uludag (2019)

These promises, however, do not come without challenges (Dumoulin et al. 2018; Kashyap et al. 2017; Yacoub et al. 2018). One dominant challenge of laminar fMRI relates to the organization of the vasculature across cortical depth (see Fig. 1). Blood flows from pial and diving arteries and arterioles to the capillary bed that directly interfaces with neuronal tissue, and is then drained via venules and ascending veins to larger veins at the cortical surface (Duvernoy et al. 1981; Turner 2002). This results in a directional blood collection -also called blood pooling- across cortical depth, towards the cortical surface. Therefore, the fMRI signal at a specific cortical depth does not only consist of measurements of the hemodynamic consequences of local neuronal activity (Heeger et al. 2000; Logothetis 2002), but also of hemodynamic changes at underlying cortical depths, closer to the gray-white matter surface. The weighting of these two factors—local neuronal activity in micro-vessels, and draining effects from micro- and macro-vasculature- is dependent upon the specific acquisition methods used (Kim and Ogawa 2012; Petridou and Siero 2017). Detailed models of the vascular architecture across cortical depth (see e.g. Boas et al. 2008; Reichold et al. 2009) additionally show that blood pooling effects might differentially affect blood oxygenation-dependent (BOLD) signals at deeper cortical depths compared to ones closer to the cortical surface (Heinzle et al. 2016; Stephan et al. 2019; Uludağ and Blinder 2018). While draining effects across cortical depth pose a challenge to laminar fMRI, these effects are largely irrelevant for fMRI at conventional resolutions (> > 1 mm^3^), as one voxel at these resolutions typically spans (most of) the thickness of the cortex.

One of the major assumptions underlying nearly all fMRI data-analysis techniques, is that fMRI responses are linearly proportional to local average neuronal activity over a period of time (Boynton et al. 1996, 2012; Cohen 1997; Friston et al. 1994; Heeger et al. 2000; Miezin et al. 2000). For this linearity to hold, two assumptions must be met: linear scaling, and temporal additivity. These assumptions largely hold for conventional-resolution fMRI, provided that the stimuli used are within a defined range of stimulus parameters that is commonly used in neuroimaging experiments (see e.g. Boynton et al. 1996, 2012; Cohen 1997; Friston et al. 1994; Heeger et al. 2000; Miezin et al. 2000). However, because of draining effects across cortical depth, laminar BOLD fMRI might violate linearity assumptions, as fMRI signals at different cortical depths are not independent. While we have previously shown that the linear scaling assumption across cortical depth holds for gradient echo (GRE) BOLD in the visual cortex (van Dijk et al. 2020), the temporal additivity assumption remains to be evaluated.

Here we evaluate whether the temporal additivity assumption for a linear system holds in human visual cortex using GRE-BOLD at 7 T. We thus seek to expand the evaluation of the GRE-BOLD amplitude linearity assumptions across cortical depth. We used three different stimulus presentation durations (2.6, 5.2, and 10.4 s, sinewave gratings at 50% luminance contrast) to elicit neuronal responses in striate and extrastriate cortex. We find that peak response amplitudes for each stimulus presentation duration increase towards the cortical surface. Additionally, we find larger response amplitudes for longer stimulus presentations. Predictions of responses to longer stimulus presentation durations based on shorter stimulus presentation durations correlated well with the observed responses, and were consistent across cortical depth. We conclude that the temporal additivity assumption holds across cortical depth for laminar GRE-BOLD fMRI in visual cortex, in line with conventional-resolution (non-laminar) fMRI.

## Methods

### Theory

We can define a system with a hemodynamic transform $L$, neuronal response over time $n(t)$, and hemodynamic response $H(t)$ (measured BOLD signal) as output:

1 $$H\left( t \right) = L\left[ {n\left( t \right)} \right]$$

While this formulation holds when responses of voxels are independent of each other, draining effects need to be considered for voxels at different cortical depths. The hemodynamic response at a given depth $y$ does not only depend on the local neuronal response, but also on responses at cortical depths closer to the gray-white matter border due to blood pooling. Thus, the measured BOLD response at cortical depth $y$ can be formulated as (Eq. 2; after Havlicek and Uludag 2019):

2 $$H\left( {y,t} \right) = L\left[ {n\left( t \right)} \right]_{y} + \mathop \smallint \limits_{{y_{0} }}^{y} w_{y} H\left( {y,t} \right)dy$$

Here $H\left(y,t\right)$ represents the measured signal over time at depth $y$; ${L\left[n\left(t\right)\right]}_{y}$ represents the hemodynamic transform of the local neuronal response at depth $y$; and ${\int }_{{y}_{0}}^{y}{w}_{y}H\left(y,t\right)dy$ represents the sum of draining contributions from the hemodynamic responses $H(y,t)$ at all cortical depths between the gray-white matter border ${y}_{0}$ and cortical depth $y$. These responses are weighted by a depth-dependent factor ${w}_{y}$. This weighting factor ${w}_{y}$ represents an estimation of the draining of altered deoxyhemoglobin content and increased blood pooling from deeper layers (Marquardt et al. 2018).

For the property of temporal additivity to hold for conventional-resolution fMRI, the response to the sum of two inputs should be equal to the sum of the responses to each separate input. Given two inputs ${n}_{1}\left(t\right)$ and ${n}_{2}\left(t\right)$, this is formalized as:

3 $$L\left[ {n_{1} \left( t \right) + n_{2} \left( t \right)\left] { = L} \right[n_{1} \left( t \right)\left] { + L} \right[n_{2} \left( t \right)} \right]$$

The easiest way to visualize this property is by imagining two neuronal responses that are elicited close in time to each other, such that the hemodynamic response associated with the first neuronal response has not yet returned to baseline when the second neuronal response is elicited. The temporal additivity assumption then states that the second hemodynamic response-resulting from the second neuronal response-simply adds to the first hemodynamic response, without interacting in any way (Boynton et al. 2012).

For laminar fMRI, draining contributions from each hemodynamic response have to be included, resulting in the following formulation of the temporal additivity assumption:

4 $$L[n_{1} \left( t \right) + n_{2} \left( t \right)]_{y} = L\left[ {n_{1} \left( t \right)} \right]_{y} + L\left[ {n_{2} \left( t \right)} \right] _{y} + \mathop \smallint \limits_{{y_{0} }}^{y} (u_{y} H_{1} \left( {y,t} \right) + v_{y} H_{2} \left( {y,t} \right) - w_{y} H_{1,2} \left( {y,t} \right))dy$$

where $L[{n}_{1}\left(t\right)+{n}_{2}{\left(t\right)]}_{y}$ is the hemodynamic response to a combination of stimulus 1 and 2 at depth$y$; ${L[{n}_{1}\left(t\right)]}_{y}$ and ${L[{n}_{2}\left(t\right)]}_{y}$ are the hemodynamic response to stimulus 1 and 2 respectively at depth$y$. The factor $\mathop \smallint \limits_{{y_{0} }}^{y} \left( {u_{y} H_{1} \left( {y,t} \right) + v_{y} H_{2} \left( {y,t} \right) - w_{y} H_{1,2} \left( {y,t} \right)} \right)dy$ represents the draining contributions from the individual and combined hemodynamic responses ${H}_{1}\left(y,t\right)$, ${ H}_{2}\left(y,t\right)$, and ${H}_{\text{1,2}}(y,t)$ at all cortical depths between the gray-white matter border ${y}_{0}$ and cortical depth $y$. These responses are weighted by depth-dependent factors ${u}_{y}$, ${v}_{y}$, ${w}_{y}$ respectively (see Eq. 2). These draining effects might not be identical for responses to the combination of two stimuli, versus the two stimulus responses separately, and thus might violate the temporal additivity assumption at a given cortical depth. However, given that temporal additivity largely holds for conventional-resolution fMRI, it can reasonably be expected that these draining effects are identical and thus cancel out, or are at least constant across cortical depth.

For the temporal additivity assumption across cortical depth to hold, BOLD responses to longer stimulus presentation durations should be predicted by temporal summation of the BOLD responses to shorter stimulus presentation durations. This should hold equally well at all cortical depths. Note that this does not necessitate that the predicted responses at a given depth need to exactly match the corresponding observed response, but merely that the goodness of fit of these predictions is constant across cortical depth. Indeed, previous research has shown that predictions built from temporally shifted and summed responses, do not correspond one-to-one to the observed responses (see e.g. Boynton et al. 1996).

### Subjects

Five subjects (all male, age range 25–45) participated in the experiment. All subjects were familiar with the MRI environment and had participated in previous experiments. They had normal or corrected-to-normal visual acuity. One subject was excluded because of poor signal amplitude, as average responses in primary visual cortex (V1) to the longest (strongest) stimulus presentation reached a maximum of 0.5% BOLD signal change, while all other subjects showed at least a 1% BOLD signal change. Signed informed consent was acquired from all subjects. All experimental procedures were conducted in accordance to the Declaration of Helsinki and approved by the ethics committee of the University Medical Center Utrecht.

### Stimuli

Stimuli were presented on a 32-inch LCD screen, specifically designed for use in an MRI environment (Cambridge Research Systems 2012). The screen resolution was 1920 × 1080 pixels, with a screen size of 69.8 × 39.3 cm, a refresh rate of 120 Hz, and a built-in linear luminance look-up table. The display was positioned at the far end of the bore and viewed via a mirror positioned in the MRI head coil. The total viewing distance was 220 cm. The total stimulus diameter subtended 10.2 degrees of visual angle (°).

All stimuli were generated in MATLAB (Mathworks 2015b) using the Psychophysics Toolbox (Brainard 1997; Pelli 1997). A fixation dot (0.1° in diameter) was presented at all times in the middle of the screen. This fixation dot was either red or green, with a color change occurring on average every 1.5 s. The stimuli consisted of sinewave gratings (0.5 cycles per degree, luminance contrast 50%) oriented in any of 4 possible directions (0–135° in 45° steps; similar to Masuda et al. 2010; van Dijk et al. 2020), restricted by a 5.1° radius circular aperture. The rest of the screen displayed mean luminance at all times. The gratings were moving perpendicular to the orientation of the bars at 1.76° per second. Movement was either towards the left or towards the right edge of the screen.

Stimuli were presented for 2.6 s, 5.2 s, and 10.4 s blocks, counterbalanced in order, and interleaved with on average 15.6 s-long mean luminance blocks. During stimulus presentation, stimuli were shown each time for 700 ms, followed by 167 ms of mean luminance. No two subsequent stimulus presentations had the same motion direction or orientation. On half the stimulus blocks, stimulus onsets were jittered by 1.3 s. A total of 12 stimulus blocks (four of each duration) were presented per run. Each run started with one block of mean luminance.

### Task

All subjects completed one session of 8 runs. Subjects were instructed to fixate on the fixation dot in the middle of the screen and report any color change of this fixation dot by means of a button press. Average fixation task performance was 78% correct (range 49–100% for individual runs).

### Visual Field Map Definition

**Fig. 2:**
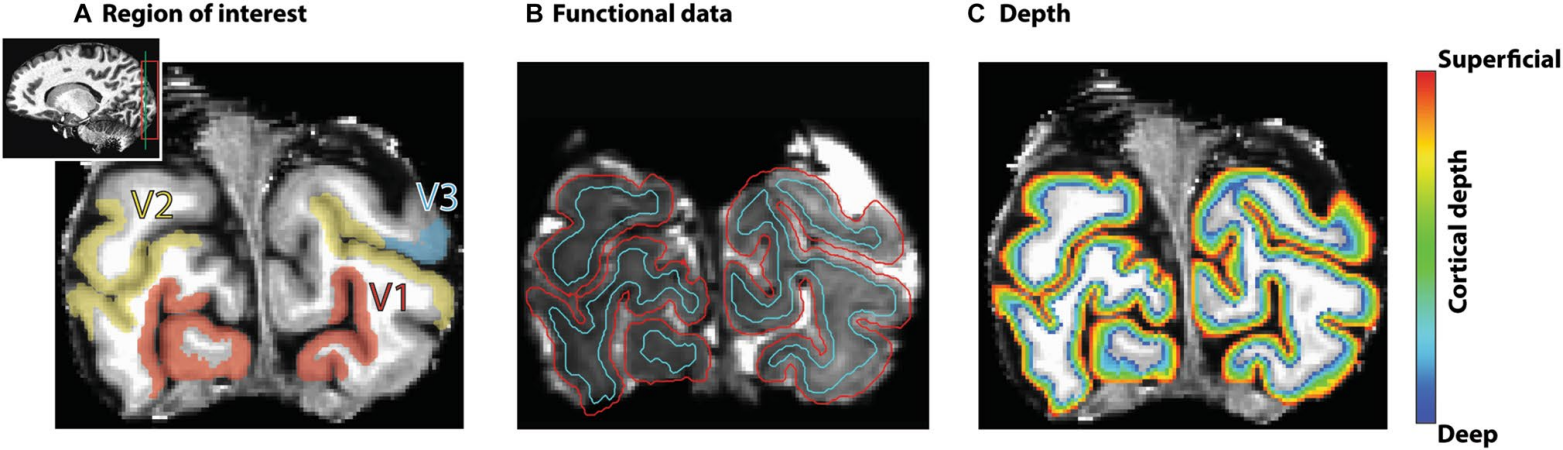
Processing steps. **a** Example region of interest definitions (V1, V2 on both hemispheres; V3 on a single hemisphere) on a coronal anatomical slice. Inset: whole brain with coverage of functional acquisition (red box) and location of the coronal slice (green line). **b** Motion- and susceptibility-corrected functional data overlaid with anatomical boundaries. Red lines denote the gray matter/CSF border while cyan lines denote the gray/white matter border. **c** Depth map for the coronal slice as in **a** and **b**

Visual field map definitions were acquired in separate scanning sessions for each subject. Procedures for this were near-identical to previous studies (e.g. Dumoulin and Wandell 2008). All data were collected using a Philips Achieva 7 T scanner (Philips, Best, the Netherlands). Details for the visual field map definitions for these specific subjects can be found in van Dijk et al. (2020), corresponding to S1, S3–S5 in this article. In short, visual field map definitions were acquired using a traversing bar-stimulus, with a 2D-EPI sequence at an isotropic resolution of 1.8 mm. Three mapping runs were collected for three subjects, while seven runs were acquired for the fourth subject. All visual field maps were restricted to 5.1 degrees eccentricity. Only V1, V2, and V3 were included in this study (see Fig. 2a for example visual field map definitions).

### MRI and fMRI Acquisition

High resolution anatomical and functional data were acquired using a Philips Achieva 7 T scanner with a maximum gradient strength of 40 mT/m and a slew rate of 200 T/m/s (Philips, Best, The Netherlands). A dual-channel volume transmit coil was used for all scans (Nova Medical, MA, USA). A 32-channel receive head coil (Nova Medical, MA, USA) was used for all anatomical scans, and two custom-built 16-channel high-density surface receive arrays were used for all functional scans (Petridou et al. 2013; MRCoils BV). These surface arrays were positioned adjacent so that the two arrays touched each other lengthwise but did not overlap. Subjects were positioned such that their external occipital protuberance was approximately aligned with the center between the arrays, at the height of the most distal receive elements from the isocenter.

Anatomical data were acquired using an MP2RAGE sequence (Marques et al. 2010). Sequence parameters were: TI1 = 800 ms, TI2 = 2700 ms, TRMP2RAGE = 5500 ms, TR/TE = 6.2/2.3 ms, flip angle α1 = 7, and α2 = 5, bandwidth = 403.7 Hz/pixel, acceleration factor using SENSE encoding = 3.5 × 1.3 (right–left and anterior–posterior respectively), resolution = 0.64 mm isotropic, whole-brain coverage, with a total scan time of 9 min 57 s.

Functional data were acquired using a T2*-weighted 3-dimensional multi-shot EPI (3D-EPI, Petridou et al. 2013; Poser et al. 2010; Van Der Zwaag et al. 2012), two shots per slice, 25 slices, 50 shots overall). The sequence parameters were: TR/TE = 59/28 ms, flip angle (Ernst angle for the used TR): 20°, acceleration factor using SENSE encoding: 2.9 (right–left) × 1.3 (anterior–posterior), number of echoes per shot: 27, voxel size = 0.80 mm isotropic, FOV = 126.3 (right–left) × 120 (feet-head) × 20 (anterior–posterior) mm, 25 coronal slices, and 10% oversampling in the slice direction. The volume repetition time was 2.6 s. Each functional run was 102 time frames (4 min 25.2 s) in duration. The acquisition volume orientation was parallel to the receive arrays.

For each functional run, we acquired an additional five volumes with reversed right-left phase-encoding. Reversing the phase-encoding direction ensured that these so-called ‘topup’ runs had the opposite geometrical distortions to the original volumes (Andersson et al. 2003; Andersson and Sotiropoulos 2016). These topup runs were used in conjunction with the original data to estimate a nonlinear warp field to create a susceptibility distortion-free volume, which is the midpoint between the functional and topup data (Andersson et al. 2003). This undistorted volume then makes it possible to align the functional data to the subject anatomy more accurately, needed for region of interest definition and cortical depth-based analysis.

### Pre-processing: Functional Data

Data processing was performed using AFNI (Cox 1996) and MATLAB (Mathworks, version 2015b). First, a warp field to correct for susceptibility distortions was calculated using a nonlinear transformation, with the first five volumes of each of the functional and topup runs as input. Motion parameters within runs were estimated by aligning each volume within a run to the first volume of that run. Subsequently, motion parameters between runs were estimated by aligning the first volume of each run to the first volume of the first run using Fourier interpolation. Next, all runs were individually despiked, scaled, and detrended. Despiking was performed using the AFNI function 3dDespike. This step was implemented to remove spurious large fluctuations of signal amplitude between two time points. These spikes were replaced with the average of the closest two non-spike time points. The scaling step entailed converting the time-series of each run to percentage BOLD. This was achieved by dividing the signal of each voxel by its temporal mean, multiplying that signal with 100, and subsequently subtracting 100 to ensure that the temporal mean of that voxel was zero percent signal change. Detrending was performed to remove slow fluctuations of the fMRI signal. For this, the AFNI function 3dDetrend with up to a fourth-degree polynomial was used. These functional runs were then temporally resampled to 1.3 s timesteps using linear resampling to match the stimulus jitter.

Next, the warp field was applied to the average over the motion-corrected, despiked, scaled, detrended, temporally resampled runs, which was subsequently collapsed over all time points to calculate the mean EPI image. This mean EPI image was then registered to the anatomy using a multi-step procedure. First, the anatomy was restricted to roughly the occipital lobe. Next, the mean EPI image and anatomy were brought into the same space by aligning the center of mass of the anatomy to the mean EPI image. Then, the ‘Nudge dataset’ AFNI plugin was used to manually shift and rotate the mean EPI image to provide a good starting point for two automated registration steps. These registration steps both consisted of affine transformations to further optimize the registration, using local Pearson correlation as cost function (Saad et al. 2010). The first transformation allowed for a maximum rotation and/or shift of 3 mm in any direction, while the second transformation allowed for a maximum rotation and/or shift of 1 mm. The transformation matrices of the manual step and the two affine transformations were combined into a single affine matrix. As a control, this matrix was then applied to the original mean EPI image to check the registration quality of this one-step procedure and ensure the correctness of the combination of the aforementioned matrices. Next, we applied the combined affine matrix and the warp field to all temporally resampled motion-corrected, despiked, scaled, and detrended runs individually to align these volumes to the registered mean EPI image. This was performed using the AFNI function 3dNwarpApply with nearest neighbor interpolation. These registered, distortion-corrected volumes were then resampled to the anatomy, resulting in the registered time-series for each run. In total, the motion-corrected, despiked, detrended time-series were spatially resampled twice, resulting in the registered, topup-corrected time-series in anatomy space (Fig. 2b).

### Pre-processing: Anatomical Data

Gray/white matter classifications of the anatomical data were carried out using MIPAV (www.mipav.cit.nih.gov/) with the CBS-tools plugin (Bazin et al. 2007; www.nitrc.org/projects/cbs-tools/), and subsequently manually optimized in 3D Slicer (Fedorov et al. 2012). Based on these corrected volumes, volume-preserving distance maps between the gray-white matter (GM/WM) border and the gray matter-cerebrospinal fluid (GM/CSF) border were computed (Waehnert et al. 2014) in 6 level-set volumes, also using MIPAV with the CBS-tools plugin. These level-sets were then projected on the distortion-corrected mean time-series for subsequent laminar analysis (Fig. 2b, c).

### Analysis

#### Temporal Response Profiles at Different Cortical Depths

For each subject we divided the distortion-corrected mean time-series into twelve blocks, each starting at stimulus onset. These blocks contained as many time points as the stimulus presentation duration (2.6, 5.2, or 10.4 s) plus a 15.6 s post-stimulus baseline. Blocks containing the same stimulus presentation duration were then averaged together. To assess the temporal responses at different cortical depths, we divided the distance map (see Pre-processing: anatomical data) into 5 depth quantiles, spanning from the GM/WM to the GM/CSF border. Next, we selected voxels within each depth bin for each visual field map (V1, V2, and V3) and calculated the mean BOLD amplitude over time for each stimulus presentation duration, along with the 95% confidence interval of the variability between stimulus blocks of the same length.

### Temporal Additivity Assessment

To assess the temporal additivity of BOLD responses for each visual field map at each depth bin, we generated predicted responses to longer stimulus presentations by temporally shifting the responses to shorter stimulus presentations, and subsequently adding this shifted response to the original response for the shorter stimulus (see e.g. Boynton et al. 1996). For example, temporally shifting a 2.6 s stimulus presentation response by 2.6 s, and subsequently adding this to the non-shifted 2.6 s response, created a predicted response to the 5.2 s stimulus presentation. These predictions were generated using the measured responses to 4 out of 8 runs. Next, we estimated the goodness of fit of these predicted responses by calculating the Pearson correlation between the predictions and the original stimulus responses of the remaining 4 runs. This procedure was repeated for each possible combination of 2 sets of 4 runs (70 in total), and the median overall fit was used for further processing (see below). To estimate the theoretical best possible fit given the data, we calculated the Pearson correlations between each pair of original stimulus responses of the first and second set of 4 out of 8 runs. This is further called the noise ceiling.

On the group level, we used the mean and 95% confidence intervals between subjects. A linear fit was calculated and evaluated for these correlations across cortical depth, for each visual field map separately. For temporal additivity across cortical depth to hold, this fit should not deviate significantly from horizontal.

## Results

### Temporal Response Profile Amplitudes Increase with Presentation Duration and Towards the Cortical Surface

**Fig. 3:**
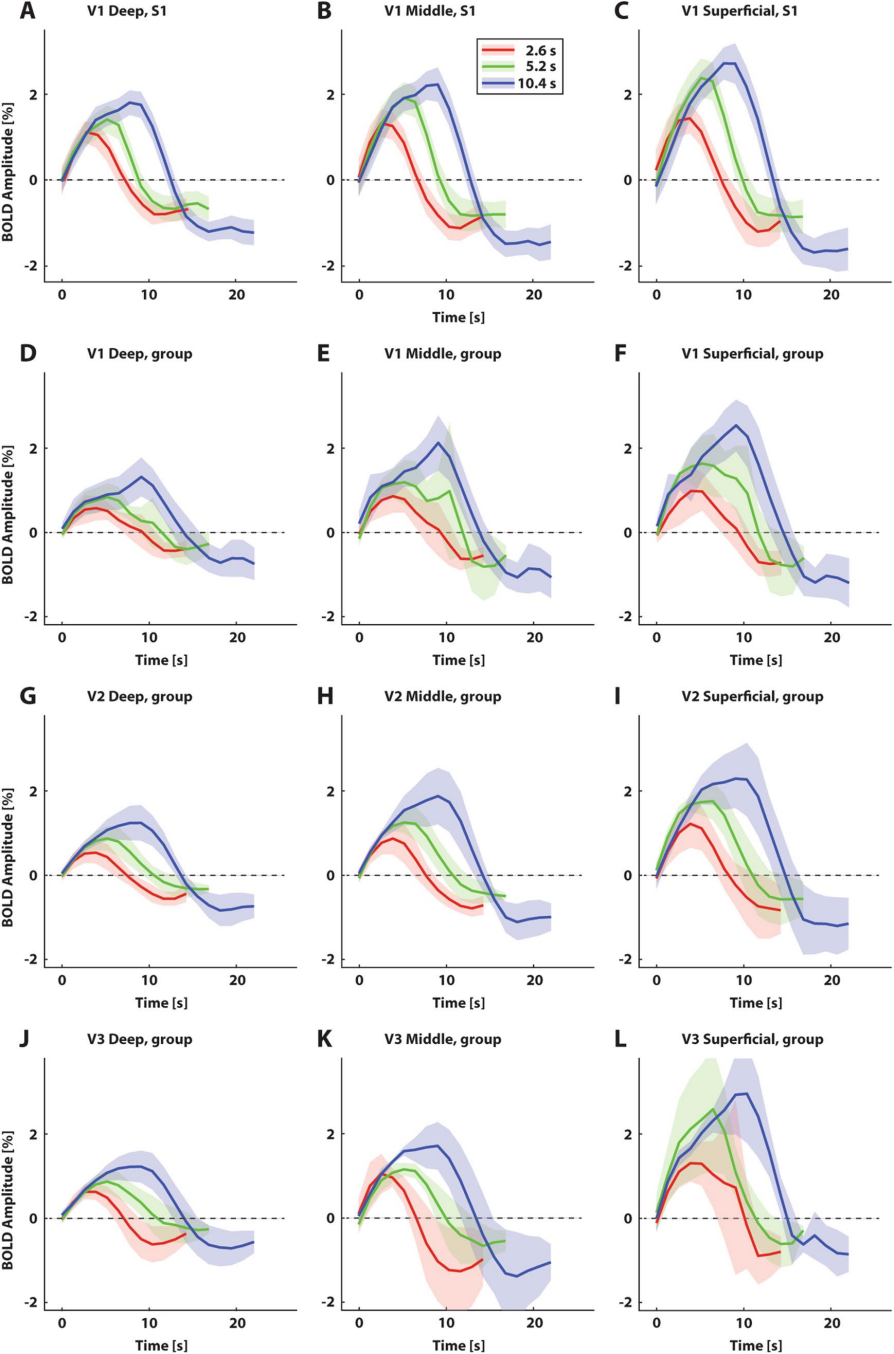
BOLD response profiles for three stimulus presentation durations at three cortical depth bins (deep, middle, and superficial). **a**–**c** BOLD response profiles for one example subject, for visual field map V1. Different colors represent different stimulus presentation durations. Shaded regions represent 95% confidence intervals of the respective means across repeated stimulus presentations. Average normalized depth: 0.13 (deep), 0.58 (middle), and 0.91 (superficial). **d**–**f** Group average BOLD response profiles for V1. Shaded regions represent 95% confidence intervals of the mean responses across subjects. Average normalized depth: 0.13 (deep), 0.59 (middle), and 0.91 (superficial). **g**–**i** As D-F, but for V2. Average normalized depth: 0.10 (deep), 0.54 (middle), and 0.90 (superficial). **j**–**l** Idem, for V3. Average normalized depth: 0.10 (deep), 0.54 (middle), and 0.90 (superficial)

For each subject, the maximum BOLD amplitude increased both as a function of stimulus presentation duration and cortical depth for all tested visual field maps (V1, V2, V3; see Fig. 3a–c for V1 data of an example subject). For the example subject, peak amplitudes in V1 at deep, middle, and superficial cortical depths for the short (2.6 s), medium (5.2 s), and long (10.4 s) stimulus presentations were: 1.13, 1.34, 1.44% BOLD (short); 1.42, 1.93, 2.38% BOLD (medium); and 1.81, 2.24, 2.72% BOLD (long). We observed similar patterns for the group averaged responses (Fig. 3d–l). Group average peak amplitudes for V1 at deep, middle, and superficial cortical depth bins for the short (2.6 s) stimulus presentation duration were: 0.58, 0.86, and 0.98% BOLD. For the medium (5.2 s) presentation duration, these numbers were: 0.85, 1.19, and 1.63% BOLD respectively. Lastly, for the long (10.4 s) presentation duration, the peak amplitudes were: 1.32, 2.13, and 2.54% BOLD. For V2, the respective peak amplitudes were 0.54, 0.88, and 1.22% BOLD (short presentation); 0.88, 1.27, 1.76% BOLD (medium presentation); and 1.25, 1.89, and 2.30% BOLD (long presentation). For V3, peak amplitudes at deep, middle, and superficial cortical depth bins were 0.62, 1.05, and 1.31% BOLD (short presentation); 0.87, 1.16, and 2.59% BOLD (medium presentation); and 1.22, 1.72, and 2.96% BOLD. The consistent increase towards the cortical surface and with visual hierarchy is in line with previous literature (see e.g. Fracasso et al. 2018; Kim and Ress 2017; Muckli et al. 2015; Polimeni et al. 2010; Siero et al. 2011). Moreover, response amplitude increased with presentation durations, likewise in line with previous research (e.g. Boynton et al. 1996).

### Shorter Presentations are Good Predictors for Longer Presentations

**Fig. 4:**
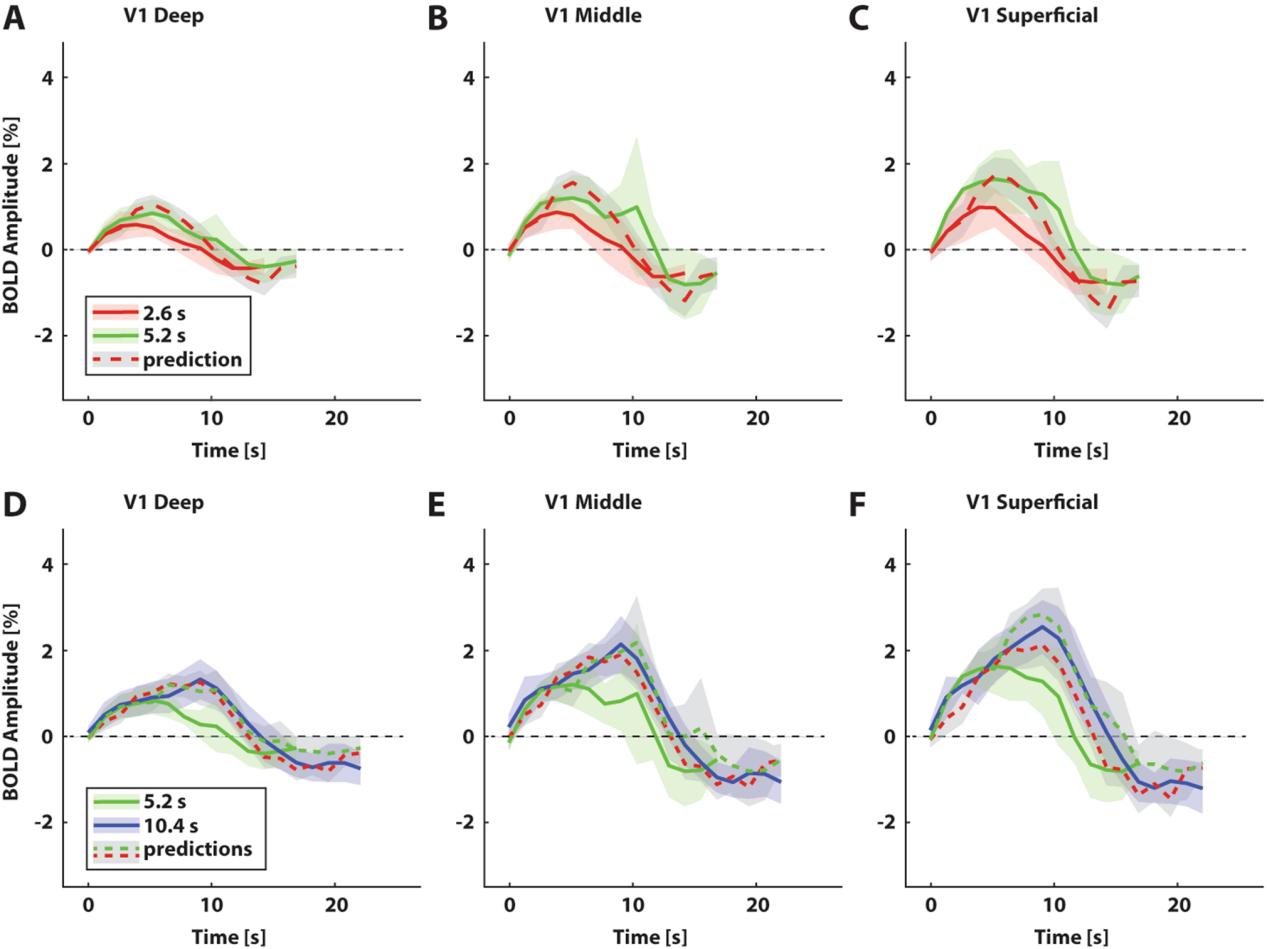
Predicted and observed BOLD response profiles at three example cortical depths for visual field map V1. Average normalized depth: 0.13 (deep), 0.59 (middle), and 0.91 (superficial). **a**–**c** Group average responses to short (2.6 s, solid red lines) and medium (5.2 s, solid green lines) stimulus presentation durations, with predicted responses to medium presentation durations (dashed red lines) calculated by shifting and summing the observed responses to the short stimulus presentation duration for each depth bin. Shaded regions represent 95% confidence intervals of the mean response across subjects. **d**–**f** Group average responses to medium (5.2 s, solid green lines) and long (10.4 s, solid blue lines) presentation durations with predicted responses to long presentation durations, calculated by shifting and summing the observed responses to the short (dotted red lines) and medium (dotted green lines) presentation durations. Shaded regions represent 95% confidence intervals of the mean response across subjects

To assess and visualize the temporal additivity assumption across cortical depth, we used responses elicited by shorter stimulus presentations to predict the responses to longer stimulus presentations. Qualitatively, the predicted 5.2 s response peak amplitudes were comparable to the observed 5.2 s responses for most cortical depths and across visual field maps (Fig. 4a–c for three example cortical depth bins for V1; see Supplementary Fig. 6A-C and Supplementary Fig. 7A-C for V2 and V3). Rise times matched well between the predicted and observed responses for all deep cortical depth bins, but were underestimated in the superficial ones. The post-stimulus undershoot was consistently overestimated.

Overall, the predicted 10.4 s BOLD response characteristics were similar to the observed 10.4 s responses in V1 (Fig. 4d–f). This included the peak amplitude, rise time and post-stimulus undershoot. The match between predicted and observed responses across cortical depth appeared to hold across visual field maps, though the match appeared qualitatively better in V1 compared to V2 or V3 (Supplementary Fig. 6D-F and Supplementary Fig. 7D-F for V2 and V3 respectively).

### Temporal Additivity Does Not Vary Across Cortical Depth

**Fig. 5:**
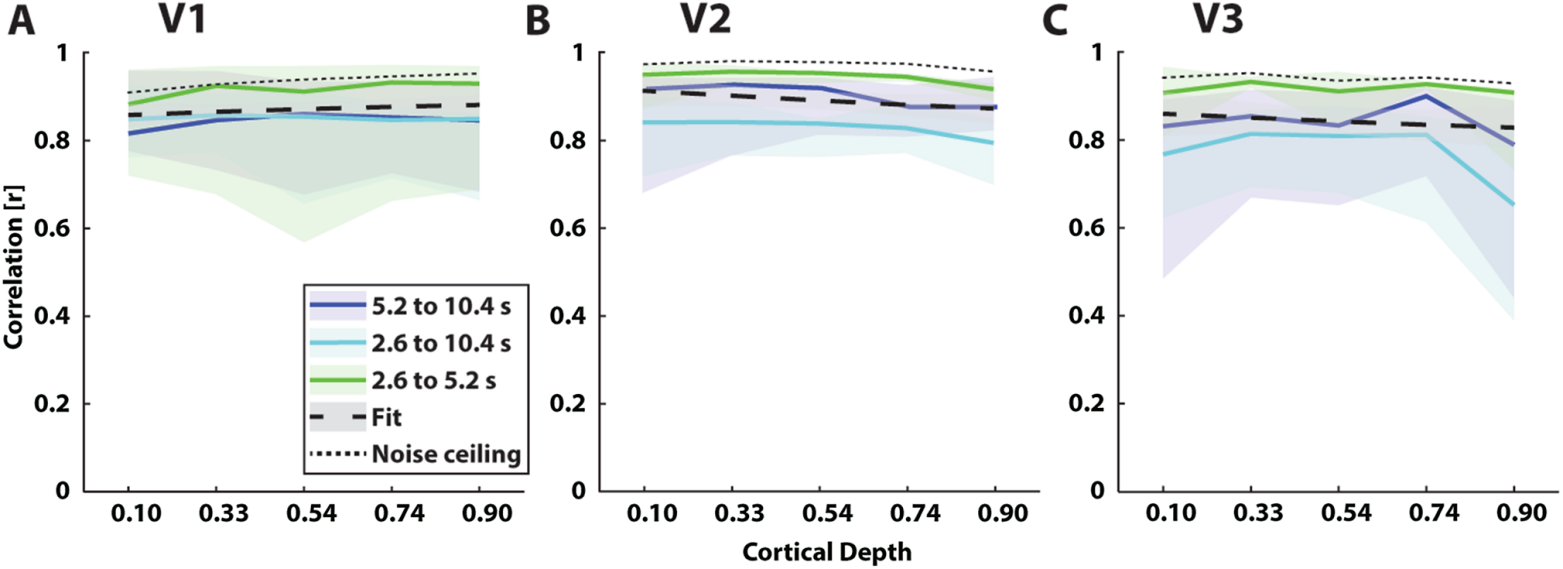
Correlations across cortical depth for visual field maps V1, V2, and V3. **a**–**c** Average correlation across scaling conditions (short presentation scaled to long and medium presentations; medium presentation scaled to long) for V1 (**a**), V2 (**b**), and V3 (**c**). Dashed black lines represent best linear fit. Dotted lines denote the noise ceiling (maximum correlation given noise in the data). Shaded regions represent 95% confidence intervals across subjects for each scaling condition

Overall, group-average predictions for V1 were good fits to their respective observed responses (Fig. 5a), with average correlations ranging from *r* = 0.82–0.93, all *p* < 0.001. The linear fit on these correlations across cortical depth did not differ significantly from horizontal: *Intercept*_*V1*_ = 0.85, *t(28)* = 58.83*, p* < 0.001, *Slope*_*V1*_ = 0.03, *t(28)* = 1.26, *p* = 0.22. Correlations for V2 ranged from *r* = 0.80–0.95, all *p* < 0.001, with no significant differences between cortical depth bins (Fig. 5b): *Intercept*_*V2*_ = 0.92, *t(28)* = 47.78*, p* < 0.001, *Slope*_*V2*_ = − 0.05, *t(28)* = − 1.56, *p* = 0.13. For V3, correlations were more variable, ranging from *r* = 0.77–0.93, all *p* < 0.001. However, the linear fit on these correlations did not differ significantly from horizontal (Fig. 5c): *Intercept*_*V3*_ = 0.87, *t(28)* = 30.42*, p* < 0.001, *Slope*_*V3*_ = − 0.04, *t(28)* = − 0.83, *p* = 0.41. Correlations were generally highest for the predictions made from the 2.6 s presentation to fit the observed 5.2 s presentation across visual field maps. Correlations for these predictions ranged from *r* = 0.88–0.96, all *p* < 0.001, and were very consistent between visual field maps and cortical depth bins. The average noise ceiling (theoretical maximum correlation) in V1 ranged from r = 0.91–0.95; in V2 from r = 0.95–0.97; and in V3 r = 0.93–0.95. Average correlations per subject can be found in Supplementary Fig. 8.

## Discussion

We assessed whether the temporal additivity assumption for a linear system holds in human visual cortex for laminar fMRI. We evaluated GRE-BOLD fMRI response amplitudes at five cortical depths, elicited by moving sine wave gratings presented for 2.6, 5.2, and 10.4 s (short, medium, and long presentation duration). We find that BOLD response amplitudes vary both as a function of cortical depth, and presentation duration, with higher BOLD amplitudes towards the cortical surface, and with increasing presentation duration. In particular, we find that shorter presentations predict longer stimulus presentations across cortical depth according to the temporal additivity principles. In conclusion, we show that the temporal additivity assumption holds across cortical depth for sub-millimeter BOLD amplitude measurements.

A growing body of research has focused on the feasibility of laminar GRE-BOLD fMRI in humans and other mammals. The majority of these studies have focused on the neurovascular coupling that underlies the fMRI signal in humans (De Martino et al. 2013; Herman et al. 2013; Huber et al. 2015, 2014; Kemper et al. 2015; Koopmans et al. 2010, 2011, 2012; Olman et al. 2012; Polimeni et al. 2010; Ress et al. 2007; Siero et al. 2011; Xing et al. 2012; Zimmermann et al. 2011); non-human primates (Chen et al. 2012; Goense et al. 2012, 2007; Goense and Logothetis 2006; Smirnakis et al. 2007; Zappe et al. 2008); and other mammals (Harel et al. 2006; Jin and Kim 2008; Lu et al. 2004; Silva and Koretsky 2002; Yang et al. 2002; Yu et al. 2014, 2012; Zhao et al. 2006). Even though relatively limited in number, the number of studies employing laminar fMRI for systems and cognitive neuroscience questions has been growing steadily in recent years (Chen et al. 2011; Cheng et al. 2001; De Martino et al. 2015; Fracasso et al. 2016a, b, c; Goodyear and Menon, 2001; Huber et al. 2015; Kok et al. 2016; Marquardt et al. 2018; Muckli et al. 2015; Smith and Muckli, 2010; Yacoub et al. 2008, 2007, for reviews see e.g. De Martino et al. 2018; Dumoulin et al. 2018; Lawrence et al. 2017; Petridou and Siero, 2017; Self and Roelfsema 2017; Stephan et al. 2019). However, most studies have interpreted BOLD amplitude measurements with caution, as these are susceptible to blood pooling effects across cortical depth since measures at different depths are inherently not independent. Thus, these studies have largely focused on non-amplitude sensitive measures. In this study, we address the temporal additivity assumption for linear systems analysis that underlies many fMRI studies, paving the way for a wider application of BOLD-based laminar fMRI amplitude measurements for systems and cognitive neuroscience.

Predictions for the medium (5.2 s) presentation based on the short presentation responses (2.6 s), consistently showed the highest correlation with observed responses of all predictions. This held true for all visual field maps and most cortical depths. This is expected, as a smaller number of time points generally results in a higher correlation. All correlations were consistently high and the trends across cortical depth were not significantly different from horizontal, indicating that the temporal additivity assumption for laminar fMRI holds across cortical depth for the tested range of stimulus parameters.

Equation 2 can be regarded as a decomposition of the more general formulation: $H\left(y,t\right)= n\left(y,t\right)*L(y,t)$. Here $H\left(y,t\right)$ is the measured local signal, $n\left(y,t\right)$ is the local neuronal response over time, convolved with $L(y,t)$, the spatiotemporal hemodynamic transform. While elegant, this formulation requires the hemodynamic transform to be defined both in space and time. In practice, most analysis methods at regular resolutions only include a temporally defined transform, as a spatially varying one is difficult to estimate. When the spatial component of the hemodynamic transform is included, it is implemented as an isotropic component (the point spread function, PSF). Due to the presence of draining veins across cortical depth, however, the spatial component of the hemodynamic transform at a laminar resolution is directional and non-isotropic. While this component has been estimated for specific laminar fMRI applications (see e.g. Havlicek and Uludag 2019), it is difficult to widely implement in practice. Our results suggest that within a cortical compartment, draining contributions from deeper cortical layers are the same regardless of presentation duration.

GRE-EPI is a widely used technique for laminar fMRI because of its high sensitivity. However, it has relatively low spatial sensitivity due to the blood draining effects (Huber et al. 2017). GRE 3D-EPI acquisitions are sensitive to both the micro-vasculature (i.e. arterioles, capillaries, and venules) and macro-vasculature (e.g. draining veins) in and around the cortex. Consequently, the observed signal is affected by blood pooling towards the cortical surface. Thus, the hemodynamic consequences of neuronal signals elicited at deeper cortical depths propagate towards the cortical surface, affecting the observed signal at more superficial cortical depths. This then leads to less laminar specificity and the regularly observed increase of BOLD signal amplitude towards the cortical surface. The increase in BOLD signal amplitude towards the cortical surface is also clearly present in the current study, with peak amplitudes for each individual presentation duration increasing in this direction. Undesired blood pooling effects across cortical depth can be dealt with in several ways. Firstly, these effects can be corrected for at the analysis level, for instance by means of spatial correction approaches (see e.g. Markuerkiaga et al. 2016; Marquardt et al. 2018), or by implementing more elaborate models of the spatiotemporal properties of the hemodynamic signal and associated dynamics of capillary, venous, and arterial effects (Aquino et al. 2012, 2014; Boas et al. 2008; Heinzle et al. 2016; Puckett et al. 2016; Uludağ and Blinder 2018). Secondly, blood pooling effects can be reduced at the acquisition level by choosing an acquisition protocol that is less sensitive to macro-vascular effects, such as spin echo-based sequences, 3D-GRASE (Feinberg and Günther 2009), or VASO (Huber et al. 2015, 2017; Jin and Kim 2008). In summary, despite blood pooling effects—which could be mitigated in several ways—temporal additivity holds for the early and intermediate visual cortex.

For the current results to be generalizable, even within the visual domain, linearity for stimuli varying in high- and other low-level domains such as spatial frequency content should also be evaluated in a similar way as presented here. Moreover, a wider range of stimulus presentation durations could be employed to better cover the extremes of this stimulus space. However, the presentation durations used here are similar to the range used in a wide range of block-design fMRI studies. Additionally, because of pooling of large numbers of measurements in this region of interest-based approach, we cannot exclude that local pockets of non-linearity might be present within a visual field map. However, our results do provide support for the temporal additivity of BOLD amplitude measures across cortical depth as a function of stimulus duration, for region of interest-based approaches. Together with our previous assessment of scaling across cortical depth (van Dijk et al. 2020), we now provide a comprehensive assessment of response linearity across cortical depth for amplitude-based GRE-BOLD laminar fMRI.

## Conclusion

We provide evidence that the temporal additivity assumption for linear systems theory is met for BOLD amplitude measures across cortical depth in V1, V2, and V3, with draining influences being constant across cortical depth. This is reflected by the similar capability across cortical depth for the shorter stimulus durations to predict the longer ones. Together with our previous work (van Dijk et al. 2020), this work provides a more complete assessment of linearity assumptions across cortical depth, and thus the validity of GLM-based analyses of BOLD amplitude measures for laminar fMRI of the early visual cortex.

## Electronic supplementary material

Below is the link to the electronic supplementary material.

Electronic supplementary material 1 (PDF 689 kb)

## Data Availability

All data and code are available from the authors upon request.
