# Supplementary material for: Validating Linear Systems Analysis for Laminar fMRI: Temporal Additivity for Stimulus Duration Manipulations
Source: Brain Topogr. 2020 Nov 18;34(1):88–101. doi: 10.1007/s10548-020-00808-y (PMC7803719; doi:10.1007/s10548-020-00808-y)
Supplement: Supplementary file 1 — Electronic supplementary material 1 (PDF 689 kb) [file 10548_2020_808_MOESM1_ESM.pdf]

Supplementary figures

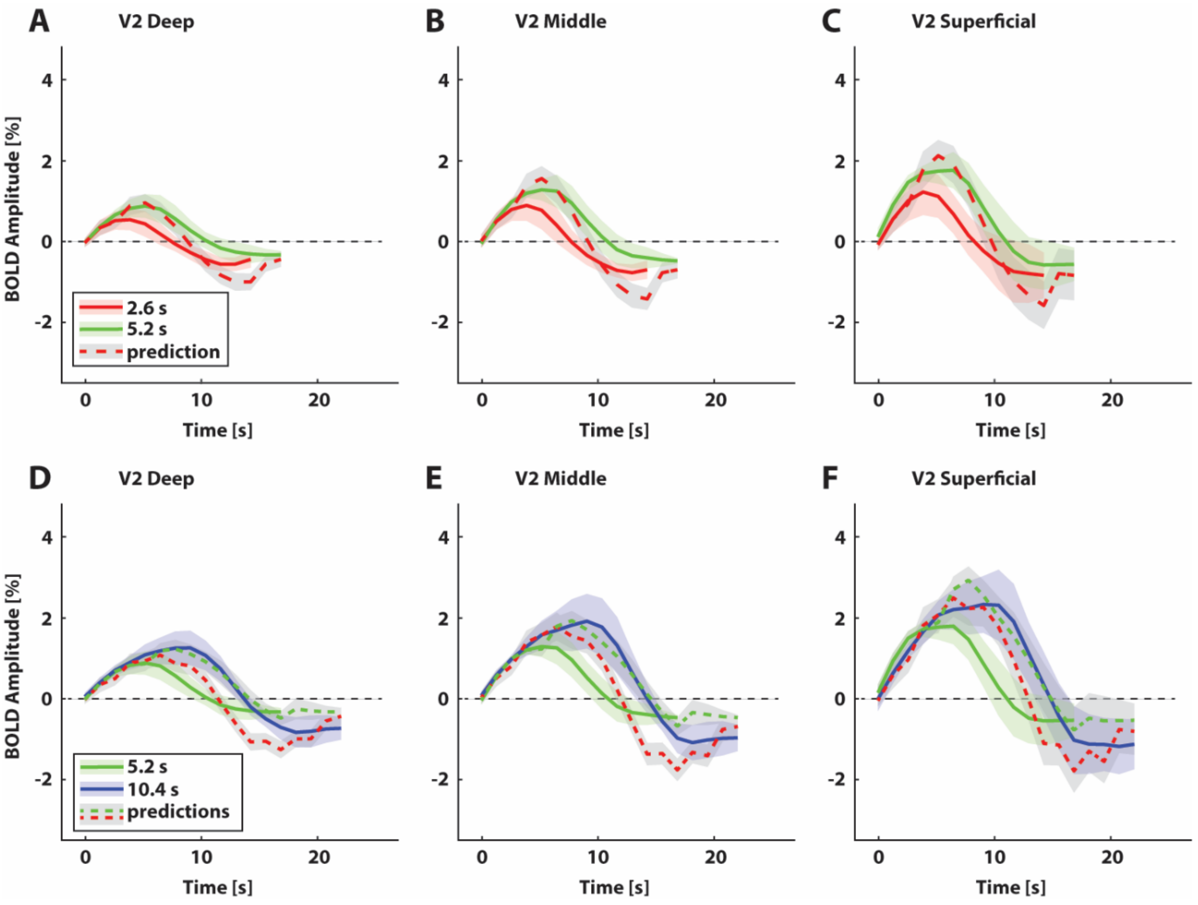

Fig. 6 Predicted and observed BOLD response profiles at three example cortical depths for visual field map V2. Average normalized depth: 0.10 (deep), 0.54 (middle), and 0.90 (superficial). A-C) Group average responses to short (2.6 s, solid red lines) and medium (5.2 s, solid green lines) stimulus presentation durations, with predicted responses to medium presentation durations (dashed red lines) calculated by shifting and summing the observed responses to the short stimulus presentation duration for each depth bin. Shaded regions represent 95% confidence intervals of the mean response across subjects. D-F) Group average responses to medium (5.2 s, solid green lines) and long (10.4 s, solid blue lines) presentation durations with predicted responses to long presentation durations, calculated by shifting and summing the observed responses to the short (dotted red lines) and medium (dotted green lines) presentation durations. Shaded regions represent 95% confidence intervals of the mean response across subjects.

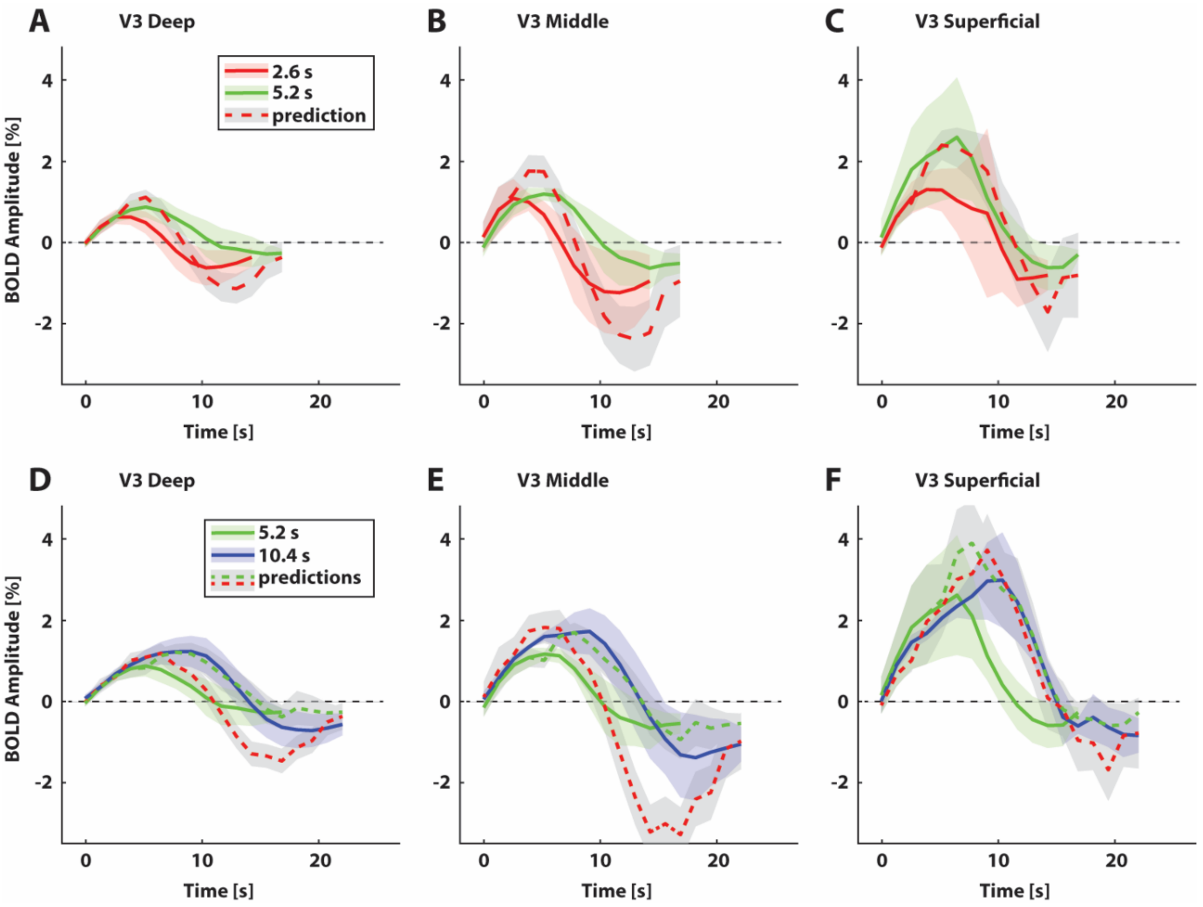

Fig. 7 Predicted and observed BOLD response profiles at three example cortical depths for visual field map V3. Average normalized depth: 0.10 (deep), 0.54 (middle), and 0.90 (superficial). A-C) Group average responses to short (2.6 s, solid red lines) and medium (5.2 s, solid green lines) stimulus presentation durations, with predicted responses to medium presentation durations (dashed red lines) calculated by shifting and summing the observed responses to the short stimulus presentation duration for each depth bin. Shaded regions represent 95% confidence intervals of the mean response across subjects. D-F) Group average responses to medium (5.2 s, solid green lines) and long (10.4 s, solid blue lines) presentation durations with predicted responses to long presentation durations, calculated by shifting and summing the observed responses to the short (dotted red lines) and medium (dotted green lines) presentation durations. Shaded regions represent 95% confidence intervals of the mean response across subjects.

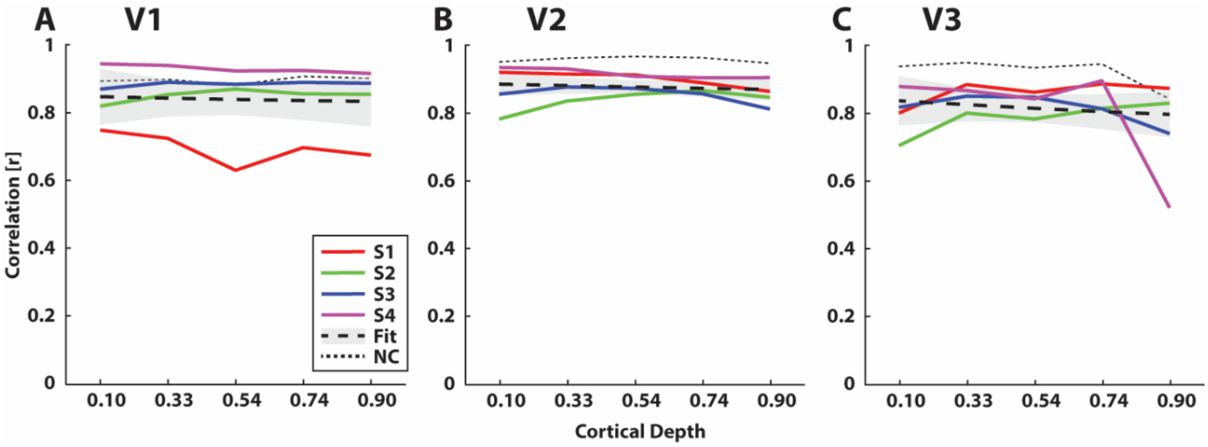

Fig.8 Correlations across cortical depth for visual field maps V1, V2, and V3. A-C) Average correlation per subject for V1 (Fig. 8A), V2 (Fig. 8B), and V3 (Fig. 8C). Dashed black lines represent best linear fit. Shaded regions represent 95% bootstrapped confidence intervals of the linear fit across subjects. Dotted black lines represent the average noise ceiling (NC) across subjects for each visual field map.
